# Broadband Full-Spectrum Raman Excitation Mapping Reveals Intricate Optoelectronic–Vibrational Resonance Structure of Chirality-Pure Single-Walled Carbon Nanotubes

**DOI:** 10.1021/acsnano.2c10524

**Published:** 2023-04-03

**Authors:** Paul Finnie, Jianying Ouyang, Jeffrey A. Fagan

**Affiliations:** † National Research Council Canada, 1200 Montreal Road, Ottawa, Ontario K1A 0R6, Canada; ‡ Materials Science and Engineering Division, National Institute of Standards and Technology (NIST), Gaithersburg, Maryland 20899, United States

**Keywords:** Raman spectroscopy, carbon nanotube, resonant excitation, Raman excitation mapping, Raman excitation profile, single-walled carbon nanotube

## Abstract

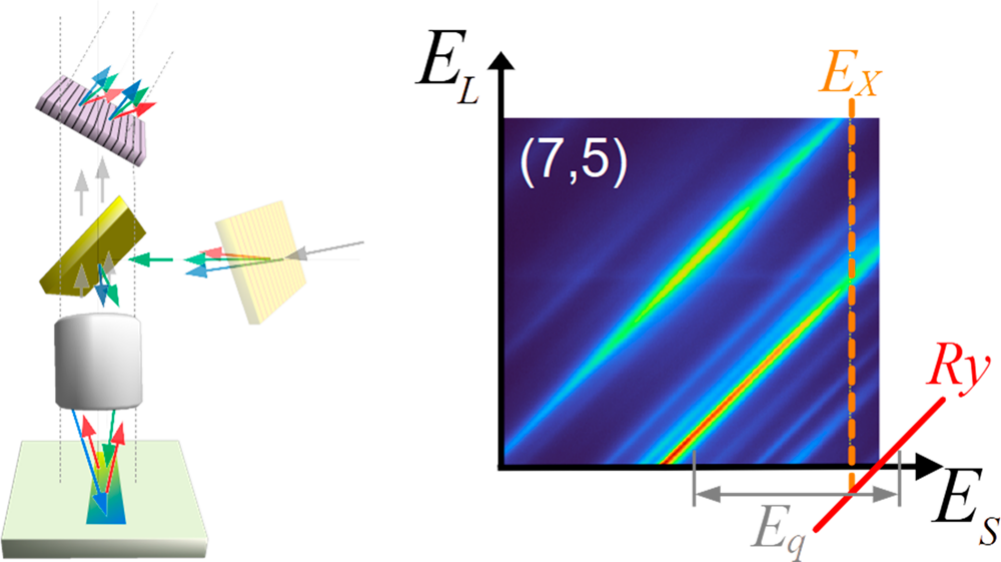

The Raman excitation spectra of chirality-pure (6,5), (7,5), and (8,3) single-walled carbon nanotubes (SWCNTs) are explored for homogeneous solid film samples over broad excitation energy and scattering energy ranges using a rapid and relatively simple full spectrum Raman excitation mapping technique. Identification of variation in scattering intensity with sample type and phonon energy related to different vibrational bands is clearly realized. Excitation profiles are found to vary strongly for different phonon modes. Some modes’ Raman excitation profiles are extracted, with the G band profile compared to earlier work. Other modes, such as the M and iTOLA modes, have quite sharp resonance profiles and strong resonances. Conventional fixed wavelength Raman spectroscopy can miss these effects on the scattering intensities entirely due to the significant intensity changes observed for small variations in excitation wavelength. Peak intensities for phonon modes traceable to a pristine carbon lattice forming a SWCNT sidewall were greater for high-crystallinity materials. In the case of highly defective SWCNTs, the scattering intensities of the G band and the defect-related D band are demonstrated to be affected both in absolute intensities and in relative ratio, with the ratio that would be measured by single wavelength Raman scattering dependent on the excitation wavelength due to differences in the resonance energy profiles of the two bands. Lastly it is shown that the approach of this contribution yields a clear path toward increasing the rigor and quantification of resonance Raman scattering intensity measurements through tractable corrections of excitation and emission side variations in efficiency with excitation wavelength.

Single-walled carbon nanotubes (SWCNTs) are a well-known family of pure carbon polymers having a planar *sp*^2^ (graphene) lattice rolled into a seamless tube with a single wall of a given diameter (*d*), chiral angle (θ), and also handedness (i.e., right-handed (*R*) or left-handed (*L*)). For perfect tubes, the possible combinations of *d* and θ are each distinct chemical species, often called chiralities, and are enumerated by the indices (*n,m*), in which *n* and *m* are whole numbers, corresponding to the vector of the superimposed hexagonal *sp*^2^ lattice.^1,2^

Real SWCNTs, however, are never entirely perfect or isolated in empty space free from environmental effects.^3,4^ They have ends, may have lattice defects, may encapsulate molecules, typically have adsorbates or surrounding molecules on their outer surface, and are frequently embedded in a liquid or solid matrix. The small size and large specific surface area of SWCNTs mean that these effects are often significant. In most samples there are variations both in the present species of SWNCTs and in their diversity of possible situations—such samples are described as “polydisperse”. Selective and sensitive characterization techniques such as optical spectroscopy^2^ are important to untangle each of these variations and to attribute observed behaviors to intrinsic material properties.

Optical spectroscopy can be used to identify the presence of specific (*n*,*m*) SWCNTs, to estimate abundances, and to reveal the physical and chemical variations for differing (*n*,*m*) and for “extrinsic” imperfections and effects.^2,5^ Raman scattering (RS) spectroscopy^6^ is one of the most important optical techniques for nanotube characterization and metrology.^7,5,4^ Most commonly this is used to identify those SWCNT diameters and (*n*,*m*) species present in a sample by comparison to a so-called Kataura plot which graphs the laser wavelength of peak resonance intensity versus the frequency of the radial breathing mode (RBM), a particular phonon mode.^2,4,7^ It is also possible to evaluate handedness of SWCNTs by Raman scattering using Raman optical activity (ROA),^8^ but circular dichroism (CD) is much more established.^9^ However, we do not evaluate handedness here.

The underlying physics of the Kataura plot relies on quantum mechanical resonance effects whereby the Raman scattering intensity of a vibrational mode is strongly enhanced near an optoelectronic resonance (electronic states or excitonic states). If the exciting laser is far from any real optoelectronic resonances, then any Raman scattering is considered nonresonant. Such scattering is weak and decreases steadily as the laser wavelength increases.^6^ However, SWCNTs have real excitonic states at energies of photons in ultraviolet (UV), visible, and near-infrared (NIR) wavelengths. This means the RS strength is enhanced and changes greatly with variation in exciting laser wavelength.^7,10^ The quantum mechanical coupling also varies strongly with the scattering phonon mode. RBMs are often only strong enough to detect for laser excitation near a resonant wavelength, so observing a strong RBM at a given laser wavelength and measuring its Raman shift, which is roughly inverse with nanotube diameter, are often enough to assign the (*n*,*m*). RS also commonly characterizes crystalline defectiveness, with the D band (∼1330 cm^–1^) intensity being an indicator of crystalline disorder across all *sp*^2^-bond-based carbons from graphite to nanotubes to graphene.^11^

When RS is used for analytical chemistry more generally, there is seldom a focus on just one single mode; rather, a set of vibrational modes in the overall RS spectrum is often used as a “fingerprint” for a molecule. Including data from several excitation lines (wavelengths) together can enhance the specificity of chemical analysis and additionally improve the sensitivity in comparison to using a single line. For SWCNTs, the diameter and (*n*,*m*) dependence of various modes has been reviewed^10^ and continues to be developed for various bands.^12,13^ All modes have differentiated intensity functions (cross-section and resonance wavelengths), intrinsic line widths, and/or shape variations with (*n*.*m*) and excitation. This implies that there is further information to be gained by looking at several modes as a set.

The variation of a given RS mode with laser excitation energy (or laser wavelength) is called its Raman excitation profile^14^ (REP) and will have a peak near a real optoelectronic (*i.e.*, absorption) resonance. We call the two-dimensional heat map of the scattering intensity versus excitation and emission energies a Raman excitation map (REM). So, the RS spectra and REPs are embedded in the REM. Furthermore, because the RS and REPs are obtained simultaneously, the relative degree to which each RS band is coupled to optoelectronic REP resonances is revealed in the REM.

While it is relatively straightforward to obtain single- or few-excitation-wavelength RS, until recently it has been less common and somewhat difficult experimentally to obtain an REM. In most studies, the variation in RS with excitation energy is limited to measurements at a few fixed excitation wavelengths provided by stable and fixed energy lasers: *e.g.*, 632.8 nm excitation from a helium–neon (HeNe) laser. A detailed REM, in contrast, has usually required less common, more complex, and frequently expensive tunable lasers, as well as tunable filters^15^ or multistage spectrometer setups. To address these shortcomings, we recently proposed a different approach to obtaining broadband REMs that we call full spectrum REM (FS-REM).^16^

As will be shown below, the data within an REM of a SWCNT sample are very rich. Measurement through the broadband approach allows us to capture an overview of the intensity variation in excitation energy and Raman shift frequency structure of nearly all common Raman modes. Given the variability in the many REM features, we believe FS-REM has the potential to be a fast, highly selective, and sensitive tool for optically characterizing SWCNTs with significant potential for general applicability to other materials. In analogy, if a fixed-wavelength RS spectrum is thought of as like a “bar code”, an REM is equivalent to a “QR code”. Such data are foundational for testing models of RS *versus* experiments and importantly present a reasonable path for quantitative relative, and perhaps absolute, resonant RS intensity determination.

## Results and Discussion

**Figure 1:**
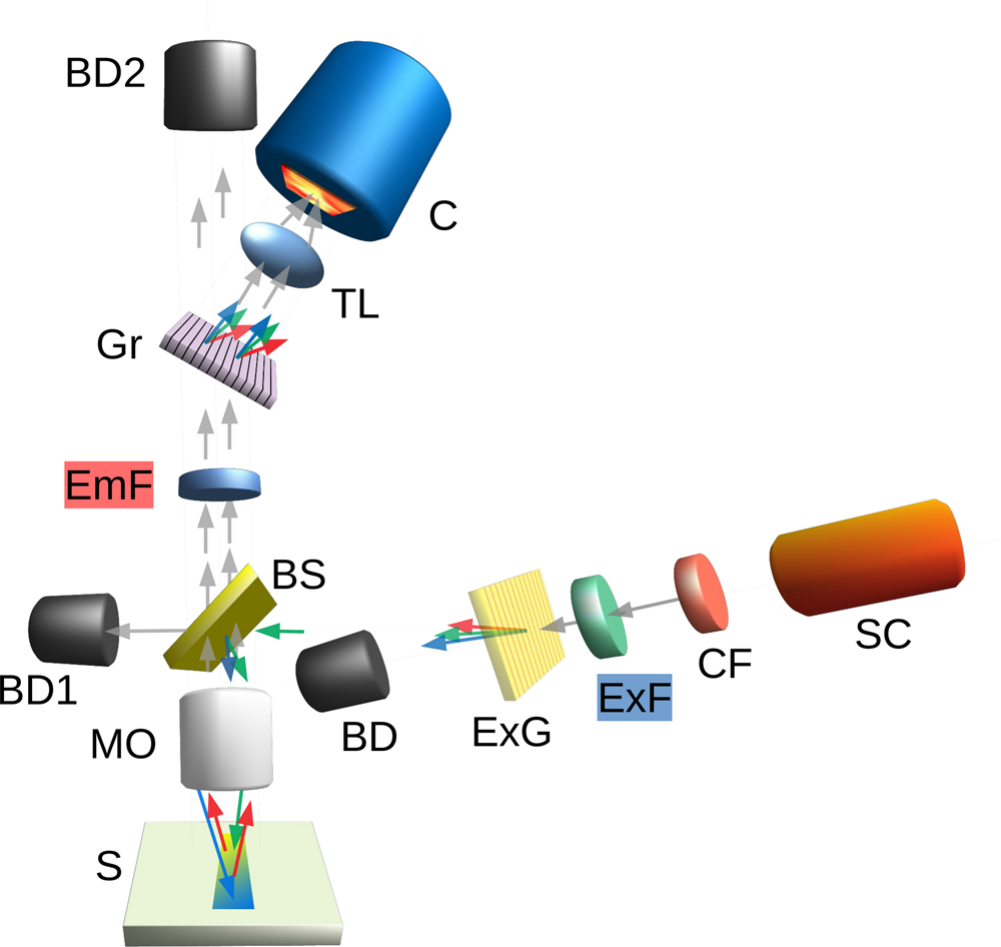
Full spectrum Raman excitation mapping epi-illumination setup. A supercontinuum (SC) light is filtered by a cleanup filter (CF) and excitation filter (ExF) before being dispersed chromatically by the excitation leg transmission grating (ExG). Unwanted light is collected in beam dumps (BDs). Illumination is reflected off a beamsplitter (BS) and focused on a sample (S) by a microscope objective (MO). This results in a rainbow line of illumination on the sample. The scattered light is collected by the same MO and passes through the BS. It is filtered by an emission filter (EmF) that is complementary to ExF. An emission transmission grating (Gr) disperses light along the perpendicular axis, and this is focused by a tube lens (TL) on an sCMOS camera (C).

A key design aspect of the FS-REM approach used here is to angularly disperse collimated white light illumination as a function of excitation wavelength (color), across a sample with a spectrally dispersive element, here a transmission grating. A schematic of the setup used in these measurements is shown in Figure 1. Experimentally, the illumination ends up focused along a line on the sample, which is color graded along its length, like a rainbow (*i.e.*, monotonic spatial variation) from which the excitation-dependent RS is spatially resolved. Overall, this is very different from building up an REM one excitation wavelength at a time through sequentially scanning the laser wavelength and adjustment of the collection optics system. The instrument used in this contribution is based on our early FS-REM instrument^16^ but with a beamsplitter placed inline to enable use of the same microscope objective for illumination and collection; we note that the use of a beamsplitter is a common configuration in conventional micro-Raman spectroscopy. We also use a lower dispersion grating to cover a broader energy range in illumination from blue wavelengths (∼480 nm) to the NIR (∼740 nm) in illumination. Matching pairs of excitation (ExF) and emission (EmF) filters were used to block the strong Rayleigh scatter, *i.e*., the direct scattering at the frequency of the excitation, while collecting RS over a broad band of wavelengths. These included a short wave pass filter in the excitation path, and a matched long wave pass filter in the emission path. Six filter pairs were used to cover the excitation range from ∼480 to ∼740 nm reported in this work.

Three highly (*n*,*m*)-pure SWCNT samples, of (7,5), (6,5), and (8,3) species, are investigated in this contribution and demonstrate the utility of the FS-REM approach. These samples were chosen because of their availability in purified form, common use within the SWCNT research community, and because they have optoelectronic resonances in the wavelength range of the illumination. The (7,5) and (6,5) species were purified with a polymer wrapping procedure carried out in toluene.^17,18^ These were then deposited by filtration on polytetrafluoroethylene (PTFE) membranes, producing uniform, opaque films of about 1 cm^2^ in area. Uniform films like these are ideal for FS-REM. Moving the line illumination across the sample produced similar Raman spectra everywhere, except where particulates caused either dark or light spots. FS-REM is possible on liquid samples; however, the chemical concentration effect that comes from depositing on a surface is beneficial to achieving a strong signal.

For the (8,3) sample an alternate sample preparation method was used due to chemical differences and a small sample volume. The (8,3) sample was prepared by aqueous two-phase extraction (ATPE)^19,20^ and consisted of approximately 250 μL of highly enriched (8,3) SWCNTs in a 10 g/L deoxycholate (DOC) in water solution. This was drop-cast on a CaF_2_ slide (see Methods). As long as the center of the spot was sampled, the measured REM was consistent and undistorted.

**Figure 2:**
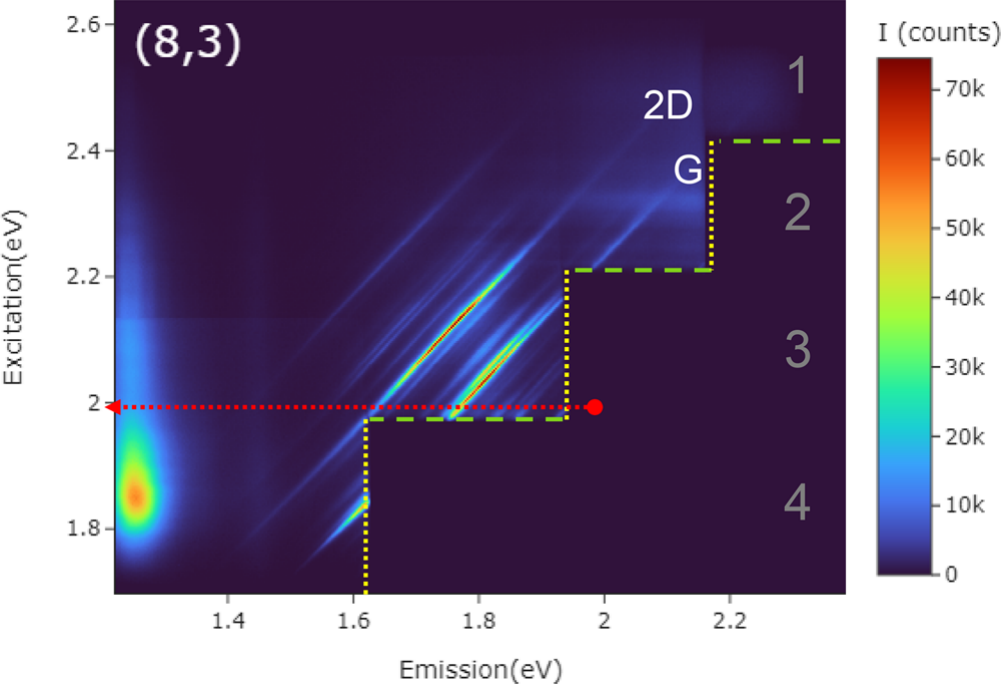
Raman excitation map for an (8,3) SWCNT. The *y* axis is the supercontinuum laser energy in eV. The *x* axis is the scattered light energy in eV. The color scale is the signal intensity in counts per pixel, here using a 30 s integration. Raman bands are streaks of near unity slope. The prominent graphitic G and 2D bands are labeled. This REM was made by combining four acquisitions with four different excitation/emission filter pairs. These are horizontal strips roughly one-fourth of the height of each map and are labeled numerically (1–4) on the top panel. The vertical yellow dotted lines show the cutoffs of the emission filters. The horizontal green dashed lines show the cutoffs of the excitation filters. The red arrow illustrates how to get a conventional fixed wavelength RS spectrum from the graph. For 2 eV laser light, the red dot is the position for Rayleigh scatter, and the (Stokes) Raman shift is the shift to the left along the dotted red arrow, so a graph of the intensity along this line is a conventional RS spectrum. The reddish spot at the bottom left is the (*E*_11_, *E*_22_) photoluminescence excitation resonance of the (8,3) SWCNT.

Figure 2 shows an experimental REM of a single species, the (8,3) nanotube, using only four of the six filter sets used in the rest of the paper. The REM is plotted in terms of energy in eV. The *y*-axis excitation energy (1.7–2.6 eV) corresponds to excitation wavelengths of 480–730 nm, and the *x*-axis is the emission energy, *i.e.*, the energy of the scattered or emitted light, and ranges from 1.3 to 2.3 eV (540 to 950 nm). Intensity in the plots is shown for energies below the Rayleigh line, including Stokes Raman scattering, *i.e.*, light scattered to smaller energy (longer wavelength) and fluorescence emission (PL) from the first excitonic optical transition (visible on the shown scale only for the (8,3) species). Plotting the figures in terms of energy is convenient because the Raman bands appear as linear streaks with a slope equal to 1—neglecting the small (<3%^10^) wavelength dispersion for most bands. The intensity of a line along its length (in eV space), parametrized by *E*_L_, provides an REP. If PL is nonresonant, it appears as a vertical line, but for resonant PL, such as the (*E*_11_, *E*_22_) excitonic transition,^21^ PL appears as a spot.^2^

The map in Figure 2 and the other maps presented below are not corrected for the variation in intensity with the color of illumination, nor are they corrected for the spectral response of the detection system. The energy-resolved spectrum of the illumination profile is shown in Figure S1 in the Supporting Information. The illumination intensity is highest at ∼1.9 eV and falls away for energies above or below this peak. The silicon detector and collection optics also have a spectral response which drops off on the low-energy side. A more complete analysis of the net spectral response of the entire system can be made by comparing it to an (ideally nonresonant) comparison material, such as highly oriented pyrolytic graphite (HOPG). Such an analysis is presented in Figures S5–S8 in the Supporting Information. Roughly speaking, the net response increases gradually (on the 100 meV scale) with increasing excitation energy from ∼1.8 to ∼2.3 eV, where it peaks and then falls off roughly linearly to zero around 2.6 eV.

In Figure 2, four pairs of matched excitation/emission filters (ExF and EmF in Figure 1) were used sequentially—short wave pass filters for the excitation and long wave pass filters for the emission. The filters were chosen so that there was essentially no overlap in transmission. Each filter pair introduces a step into the maps where the instrument is effectively blind. This is the origin of the stepped dark regions on the lower right side of the maps. The excitation filters are short wave pass filters. Their cutoffs are represented by the horizontal dashed green lines in Figure 2. The emission filters are long wave pass filters, represented by vertical yellow dotted lines in Figure 2. Obviously, the spectral features are continuous through the blocked areas, they just are not detected.

As noted earlier, when plotted in this way, Raman bands are represented by lines of unity (or at least near-unity) slope. Two prominent Raman bands, the 2D and the G band, are labeled in Figure 2. The larger the (Stokes) Raman shift, the further to the left the band will be. Before looking at the Raman bands of different (*n*,*m*) SWCNTs more closely, we consider what REMs should look in the most elementary picture of resonant RS.

**Figure 3:**
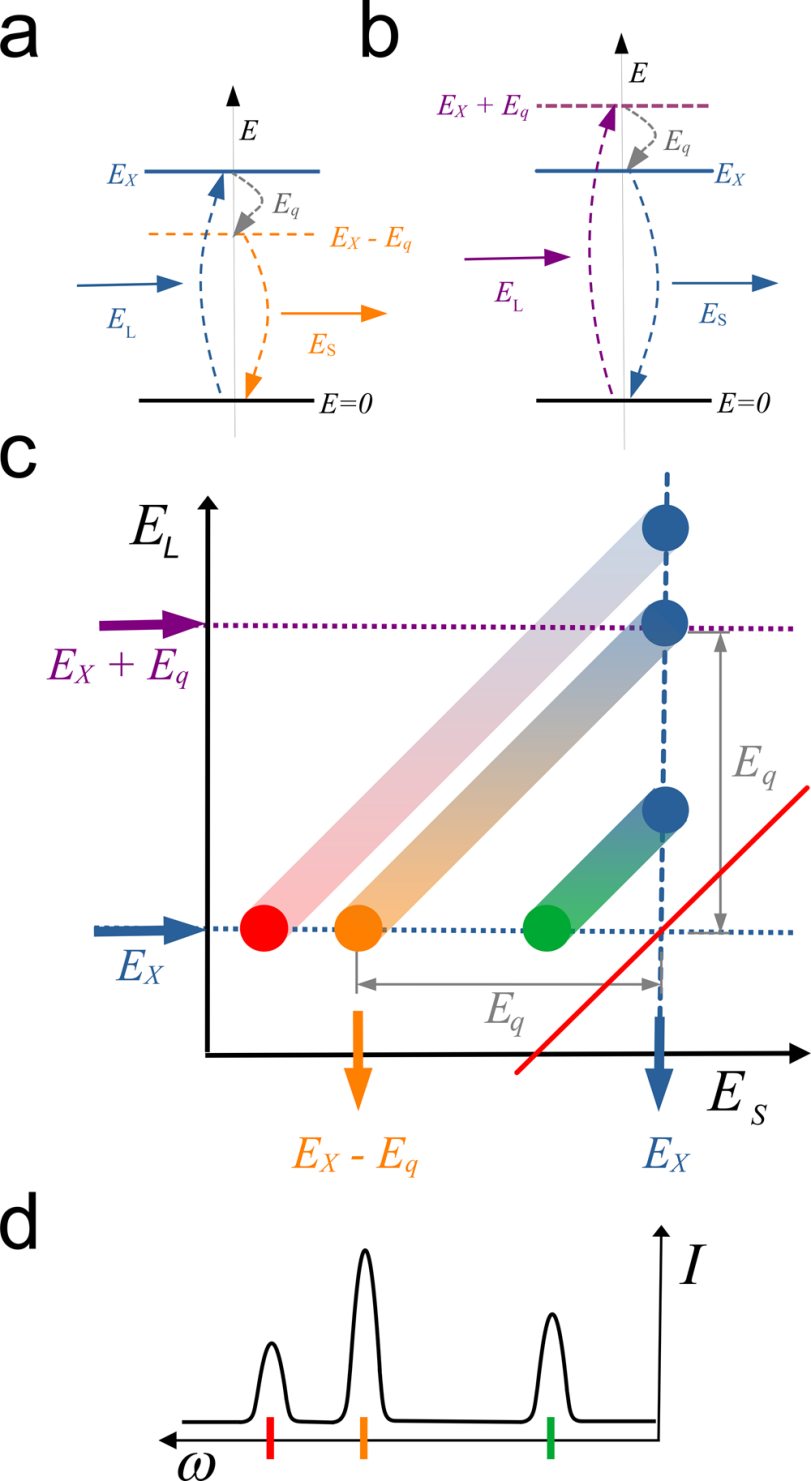
Schematic of resonance. Laser light (energy *E*_L_) is scattered to a lower energy (*E*_s_) with the emission of a phonon (*E*_q_). The electronic ground state is *E* = 0, and the excited state is *E*_X_. (a) Energy diagram of ingoing resonance. The laser is resonant with the real excited state. (b) Energy diagram of outgoing resonance. The laser is not resonant, but the scattered light is. (c) Schematic of Raman excitation map. Three different phonon modes are illustrated as diagonal bands. Ingoing resonances appear along the dotted blue line. Outgoing resonances are along the vertical dashed blue line. (d) Schematic of a conventional fixed-wavelength RS spectrum extracted along a horizontal line with intensity (*I*) as a function of Raman shift (ω) with each mode represented by a single peak.

The resonant RS process and how it is represented in REM and conventional single-wavelength RS spectroscopy is illustrated schematically in Figure 3. In RS, incident light mixes with the vibrational modes of the material or molecule, generating light which is shifted by a vibrational (phonon) energy from the incident light energy (*i.e.*, wavelength). Here, only shifts to lower energy (“Stokes”) scattering will be considered. In the case of resonant RS, real optoelectronic states are involved and this leads to greatly enhanced (up to ∼10^5^×)^6^ scattering intensities. The simplest case is shown in Figure 3a. An incident laser photon (energy *E*_L_) promotes the system in its ground state (*E* = 0) to a real excited state (*E*_X_). A phonon is created (energy *E*_q_) along with a photon scattered to reduced energy, returning the system to its ground state.

Figure 3a shows the ingoing photon resonance, because the ingoing photon matches the excited optoelectronic state. But there is always a corresponding outgoing resonance,^22^ illustrated in Figure 3b. In this case, the incident photon is above the real state, but emission of a phonon brings it down to the real state, so emission of a photon at the real state energy returns the system to its ground state. This is the outgoing resonance, and it also shows enhanced scattering intensity. For the RBM band the phonon energy is small, and the two resonances are indistinct.

The exact form of the RS intensity is complicated, though it can be simplified and modeled through a series of approximations. As explained by Duque *et al.*, a model of intensity of Raman scattering for a given single phonon mode as a function of laser energy in molecular systems generally and for the G band resonance in SWCNTs specifically is
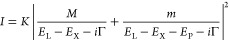
where *E*_L_ is the laser energy, *E*_P_ is the vibrational energy of the phonon, *E*_X_ is an excited optoelectronic state, Γ is a damping factor, and *M* and *m* are matrix elements for incident and scattered phonons.^22^ The origin of Γ is just the modeling of the finite lifetime and therefore spectral broadening of the states, commonly used in perturbation theory.^14,22^ The first resonance is the ingoing photon resonance, and the second is the outgoing photon resonance. These two terms add—before taking the square—to determine RS probability and so interfere. When the motion of nuclei is fully decoupled from electronics, *m* and *M* are equal, but for SWCNTs this does not match experiment and their asymmetry can be explained by coupling between electronic and nuclear degrees of freedom (*i.e.*, “breakdown of Condon approximation”).^22^ This model has successfully fit experimental data.

From this equation, the REP is Lorentzian-like for *E*_L_ close to *E*_X_ and close to *E*_X_ + *E*_P_. If the phonon energy (*E*_P_) is large enough compared to the line width, as may be the case for the G band, these resolve into two separate resonances. On the REM plot the ingoing resonances for each vibrational mode appears as spots along the horizontal line where *E*_L_ = *E*_X_. In the schematic three phonon modes are illustrated. That in the middle is a mode of vibrational energy *E*_q__._ Incoming resonances have the distinction of all lighting up at one excitation energy in this model.

The outgoing resonances appear as spots at the energy equal to each specific mode’s phonon above the Rayleigh scattering line (*E*_S_ = *E*_L__,_ the red diagonal line of unity slope). So, for example, the phonon of vibrational energy *E*_q_ produces a spot on the horizontal line at *E*_L_*= E*_X_ + *E*_q_ at the point where the scattering photon energy is the real excited state energy, *E*_S_ = *E*_X_ (the vertical dotted line). In this picture, if the vibrational modes are reasonably well spaced, only one will be resonant at any given laser energy.

Samples will have not one but various RS modes, and using this simple picture we can already understand that every vibrational mode will have its own resonance structure. Thus, the two-dimensional map of excitation wavelength versus emitted RS, either in Raman shift or in wavelength (*i.e.*, the REM), includes substantially more information than the simple Raman scattering at a fixed wavelength. This is because the REM has contributions from the mechanical vibrations and separately the optoelectronic resonances, as well as their lifetimes, and the strength of the coupling between the electronic and vibrational resonances.

Especially interesting, though, is that each ingoing and outgoing resonance is represented by a complex quantity which adds *via* a complex square. This implies that these resonances can interfere with each other. This interference is represented in the schematic by the shading of the streaks between the ingoing and outgoing resonance spots on the REM plot. It should thus be expected that there will be some interference component between the spots in an REM plot and that the spots themselves can be distorted by interference.

Duque *et al.* also highlight the effect of the asymmetry in the values of *M* and *m* on interference.^22^ In particular, that Lorentzian peaks can be distorted and drawn closer together along the diagonal line between *E*_S_ = *E*_L_ (horizontal line) and *E*_S_ = *E*_X_ (the vertical dotted line).

Figure 3d shows schematically how a conventional Raman spectrum can be extracted from the map by taking a trace of the intensity along any horizontal line, corresponding to a particular choice of laser wavelength. Here, the *x* axis is opposite to the conventional way RS spectra are plotted. Usually the amount of energy lost is plotted so a Stokes phonon energy is plotted as positive. Since we have plotted the REM simply in terms of scattered phonon energies, it makes more sense to have Stokes phonon energy going to the left of the Rayleigh line on the REM map.

**Figure 4:**
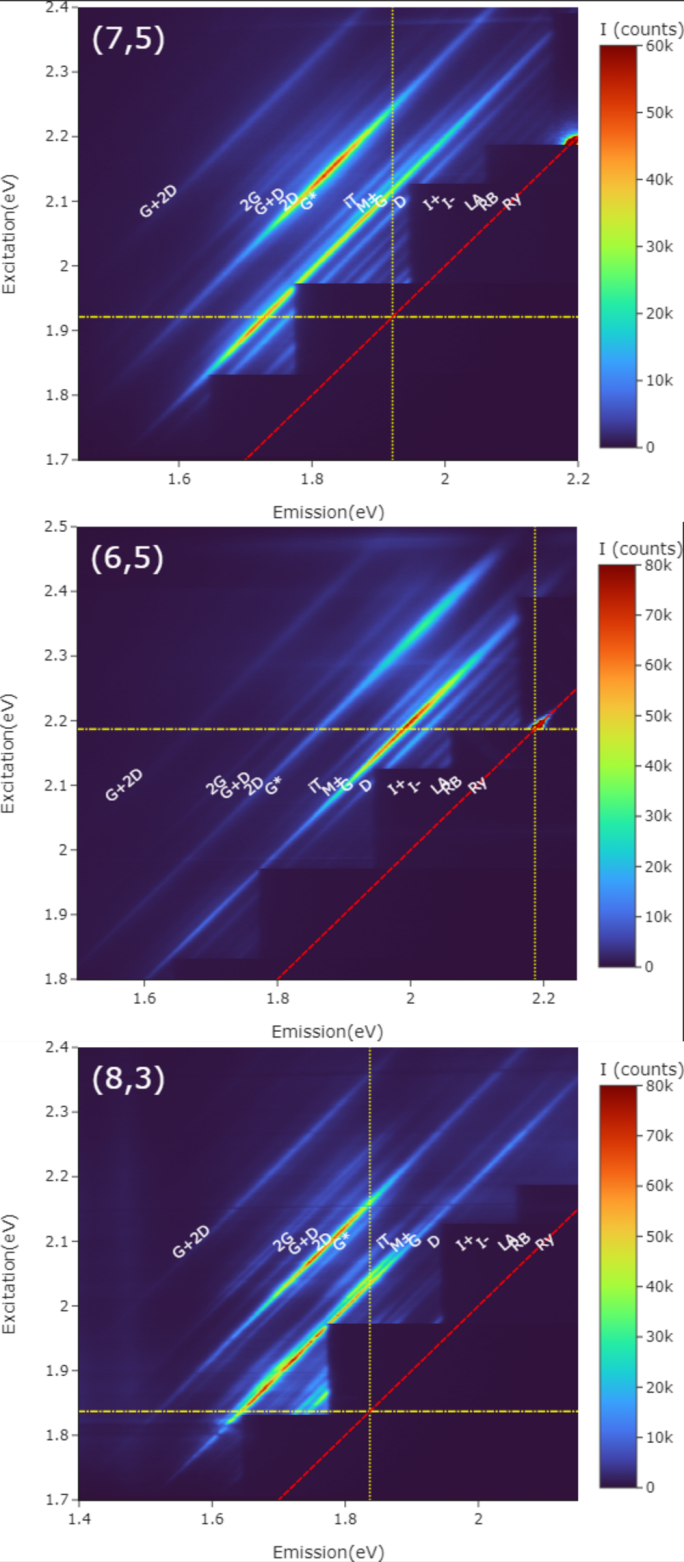
Detail of Raman excitation maps for several (*n*,*m*). Closer view of three nanotube species: (7,5), top; (6,5), middle; (8,3), bottom. Bands are labeled according to the most common nomenclature (see text for details). The excitonic resonance (*E*_22_) is marked on the excitation axis (*y* axis) by a horizontal line (yellow, dash-dotted) and the emission axis (*x* axis) by a vertical line (yellow, dotted). The diagonal line (red, dashed) marks the position of Rayleigh scattering (*i.e.*, zero Raman shift). Here six filter sets were used, with 12 s integration for each filter set. Fluctuations in the lower left of the map for the (8,3) SWCNT, seen mainly in the G band, were not systematic but were related to photoinstability.

Figure 4 shows detailed maps for several species, (7,5), (6,5), and (8,3), using all six filter combinations, enlarged slightly more than in Figure 2 and annotated to facilitate comparison to the simple picture presented in that figure. Note that the axis and scales are not the same for all plots. The modes are assigned to their most commonly used names^10^ (G, D, *etc.*) or shortened versions: Ry for Rayleigh scattering, RB for RBM, I^-^ for IFM^-^, I^+^ for IFM^+^, M± for both M bands (M^+^ and M^-^), and iT for iTOLA. The red dotted diagonal line of slope unity is the position of Rayleigh scatter. The Raman shift is the horizontal energy shift to the left of the Rayleigh line (Raman shifts are usually given in units of cm^–1^, where 1 eV = 8064 cm^–1^). The energy of the *E*_22_ exciton resonance of SWCNTs is plotted on both axes as a dotted vertical line and a dot-dashed horizontal line, using tabulated values.^21^ As noted above, these plots have not been corrected for illumination intensity variation with wavelength, or detector response. (see the Supporting Information for an evaluation of this correction). However, all share the exact same illumination and detector response such that they can be compared directly with one another.

The exciton resonance can be found directly from optical spectroscopy and is most established for photoluminescence (PLE) spectroscopy.^2^ Published tables give the *E*_11_ and *E*_22_ resonances precisely for SWCNTs in an aqueous surfactant.^21^ This is quite general down to ∼50 meV accuracy, but the particular choice of tube can leads to shifts on the scale of tens of meV.^3^ The effects of environment on Raman spectra can be fit to semiempirical models,^7,10^ but they can be determined more accurately and precisely by experimental measurement than those that can be predicted. In the case of the (8,3) SWCNT, the *E*_22_ energy can also be read off the map in Figure 2, in which the (*E*_11_, *E*_22_) PLE peak for that species is visible and this compares well with tables. The (7,5) and (6,5) surface SWCNTs did not show a distinct PLE resonance, so the tabulated position for surfactant-dispersed tubes was used, which was close to those obtained with PLE measurements for these SWCNTs in toluene solutions (see Figure S2 in the Supporting Information) and aqueous suspensions (Figure S3).^21^

Looking at the real maps, we can see how they compare to this elementary theory. (Low-energy modes get cut off intermittently by the filters, so we focus mainly on higher-energy modes.) The yellow lines mark the positions of the *E*_22_ resonances, with the horizontal line being the ingoing resonance and the vertical line being the outgoing resonance. The model predicts the Raman enhancement to be strongest along these yellow lines, with quantum interferences in the diagonal line connecting these resonances, so that scattering is expected to be enhanced in the top left quadrant made by the yellow lines and trailing off below or to the right. There is, in a broad sense, a general agreement with this picture. The strongest resonance falls, as expected, in the top left quadrant, with the most enhancement near and between the yellow lines. In all three, there is a G band maximum on or close to the yellow lines. The top (7,5) nanotube is exemplary with both the ingoing and outgoing resonances being clearly visible and generally enhanced strength between these maxima, falling off on either side. Other bands also show similar structures, though the outgoing resonances are not well captured for the (6,5) SWCNT due to the range of the instrument.

For the (7,5) SWCNT sample, the intermediate frequency modes (I^+^, I^−^), G, M bands (M^+^ and M^–^, here indistinct and so denoted collectively M±), iTOLA (here labeled iT), and 2D bands also drop off, moving to the right across the vertical yellow line. The gaps in the map mean that there is less information for the ingoing resonance, but the G band clearly trails off below the horizontal line.

The experimental (6,5) map is better situated to examine ingoing resonances. In the (6,5) SWCNT the D, M±, iTOLA, and to a lesser extent G and 2D bands drop off below the horizontal yellow line, with the M± and iTOLA bands showing a structure similar to that of the G band. Furthermore, for the (6,5) SWCNT the iTOLA, M, and D bands all show stronger intensities near the horizontal yellow line (ingoing resonance).

For the (8,3) SWCNT the G, M±, iTOLA, and G* bands are enhanced between these yellow lines with peaks close to them. Fluctuations in intensity at the bottom left of the map, near the horizontal yellow line and most prominent for the G band, come from the loss of photostability for this sample alone. Where the illumination approached this resonance, the nanotubes became irreversibly damaged. Other bands are not tracked much below the horizontal yellow line but do drop off on moving to the right of the vertical yellow line. So overall, the bands are enhanced in the top left quadrant, and especially near the yellow lines, as expected from the model.

One aspect which could possibly be a departure from the simple model is that enhancement spots are not centered on the yellow lines; rather, they are drawn in along the diagonal line toward each other. However, Duque *et al.*, found that this could be explained even in the simple model as a consequence of the asymmetry introduced by nonequal matrix element components *M* and *m*.^22^

The simple model is does surprisingly well in explaining the general features of the maps. In fact, there are real physical considerations which make it unreasonable to expect very close agreement. Importantly, most of the high-energy modes in the real REMs (the G band being an important exception) are caused by more than one phonon scattering event. This means that the scattering probability is more complicated. In addition to the effect of the combination of phonons, there is also the possibility of enhancement between them due to one of the phonons by itself scattering into a real state. The effect of that is to introduce the possibility of additional resonant enhancement at points along the diagonal of the phonon band between the yellow lines.

Furthermore, the model includes only two real optoelectronic states. In reality SWCNTs have many optoelectronic resonance levels, due to their various different excitons and also due to unbound electrons and holes. Any given phonon mode can interact with any given optoelectronic resonance to a different degree. Accounting for all these affects in detail will be challenging, but it is promising that all this information is accessible *via* experiments.

The dispersion of SWCNT Raman peaks—the change in Raman shift with laser wavelength—has been well studied for a number of bands,^1^ and while this is a promising area to explore with the full spectrum technique, the REMs here do not have high enough spectral resolution to compare precise Raman shifts. On the other hand, the resonant excitation profile (REP) for most bands has been much less studied and is well covered in these maps, and so we examine REPs below.

**Figure 5:**
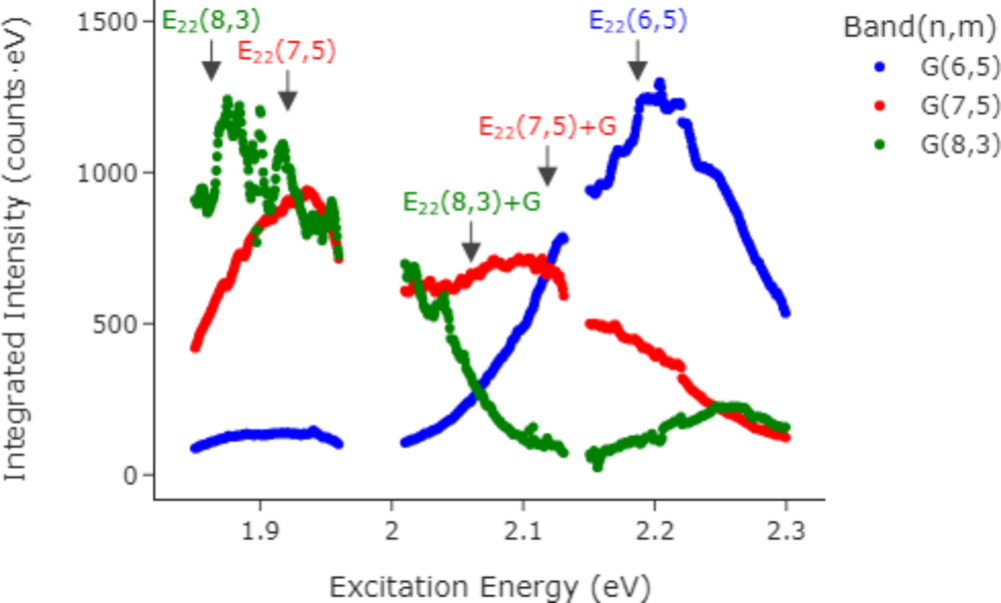
G band Raman excitation profiles. G band Raman excitation profiles (REPs) extracted from the maps of Figure 4 are shown for each species. The *E*_22_ excitonic transition position, from tables for surfactant dispersions,^21^ is marked by an arrow for each (*n*,*m*), and the position above the G band energy is labeled for the (7,5) and (8,3) SWCNTs only since the (6,5) +G resonance is off the map. The integration time was 12 s. The integrated band intensity was determined by fitting a simple, single Lorentzian to the map data at each particular excitation energy. The breaks in the lines are where the fit is poor due to the filter edges.

Probably the most explored REP is that of the G band. To compare to previous work, we extract out an integrated intensity for the G band alone by fitting the above to a single Lorentzian peak using an open-source Python peak fitting module (lmfit).^23^ The resulting integrated peak intensities are shown as a function of excitation energy in Figure 5. This graph is not corrected for the spectral profile of the illumination or the detector response; however, intensity-corrected curves, determined by using highly oriented pyrolytic graphite (HOPG) Raman bands as a reference, are included in Figure S9 in the Supporting Information.

The (7,5) G band REP can be compared directly with previous work.^22,24^ In this contribution the peaks are broader and less resolved than in previous reports; however, this likely corresponds to a shorter lifetime for the excited states in our solid sample especially as compared to those in liquid dispersions. Different samples of the same SWCNTs have shown sharper^22,24^ or less clearly resolved^25^*E*_22_ resonances. This is likely related to different sample preparations giving rise to different lifetimes. For example, the (6,5) and (7,5) here are polymer wrapped, where most prior single (*n*,*m*) studies use surfactant SWCNTs—this difference is likely to be important. Similarly, our use of dense solid films, as noted above, is a difference since most comparable single (*n*,*m*) G band REP studies used dipersions,^22,24,25^ or air.^26^ In comparison the concentration of SWCNTs in a film is very high such that interactions including resonance energy transfer (RET)^27^ or more complicated phenomena^28^ could be more significant for these dense SWCNT films.

Where G band REPs for different excitonic resonances (*E*_11_, *E*_22_) have been reported,^25^ the *E*_22_ REP has been found to be broader than the *E*_11_ REP, which is understandable as being due to the shorter lifetime of *E*_22_. A well-defined G band REP from *E*_33_ has been reported for a large-diameter air-suspended SWCNT.^29^ Air-suspended SWCNTs typically have the best optical properties, so a longer lifetime may be responsible for the sharper REP in that case. The asymmetry in the G band REP is significant.^22^ We do observe the same type of peak height asymmetry as in these references, with the lower energy resonance (*E*_22_) being higher than the higher energy resonance (*E*_22_ + G). In Figure 5 this is distorted by the spectral characteristics of the system, but the same asymmetry persists after correcting for the instrumental factors (Figure S9 in the Supporting Information).

It has also been shown experimentally that the G band REP peak for (6,5) SWCNTs in the vicinity of *E*_22_ becomes distorted by the effect of bundling.^28^ The sample preparation here is different, but the nanotubes are expected to be in close proximity and so can be considered heavily bundled. This bundle-REP effect may be the origin of the small fluctuations near the peak at 2.2 eV in the (6,5) G band REP of Figure 5.

The REP of other bands has comparatively been much less explored. Here, as is apparent from the maps of Figure 4, a number of phonon modes show interesting and intricate REP structures. Some of the modes have particularly narrow REPs, as seen in the maps of Figure 4. For the (8,3) SWCNT map the M, iTOLA and G* bands are narrow and there is evidence for two peaks, one nearer the horizontal yellow line and one nearer the vertical yellow line for M and iTOLA. The narrowness of the M and iTOLA REPs compared to the G REP is shown explicitly in Figure S12 in the Supporting Information. For the (6,5) SWCNT, the M band and iTOLA band also appear narrow and near the horizontal yellow *E*_22_ line. The narrowness of the iTOLA REP compared to the G REP for the (6,5) SWCNT is also shown explicitly in Figure S13 in the Supporting Information.

Broadly speaking, the narrowness of the REPs implies that the appearance of the conventional fixed-wavelength RS spectrum of a particular (*n*,*m*) depends significantly on the wavelength used to analyze it. Some of these peaks are sufficiently narrow that measurement by conventional fixed-wavelength RS is likely to miss their strong enhancement entirely. For example, in Figure 4, the M± bands of (8,3) exhibit a particularly sharp resonance near 605 nm, which is not especially close to any commonly used wavelength for fixed wavelength RS. (see Figure S12 in the Supporting Information for the REP).

The excitonic resonances (the real states in the simple picture) depend on the environment surrounding and inside each SWCNT, which can easily lead to shifts of 20 meV or more depending on sample preparation.^30^ Therefore, the overall spectrum of a particular SWCNT species can change substantially in different environments. It thus follows that the *resonant* RS spectrum at a fixed wavelength should not be thought of as something unchangeable; peak-to-peak Raman intensity ratios necessarily change with changing excitation wavelength. Consistent with comparisons of the RBM and G bands,^31^ in very general terms, the REP is different from band to band—sometimes the effect of resonance may be small, but sometimes it causes significant differences.

**Figure 6:**
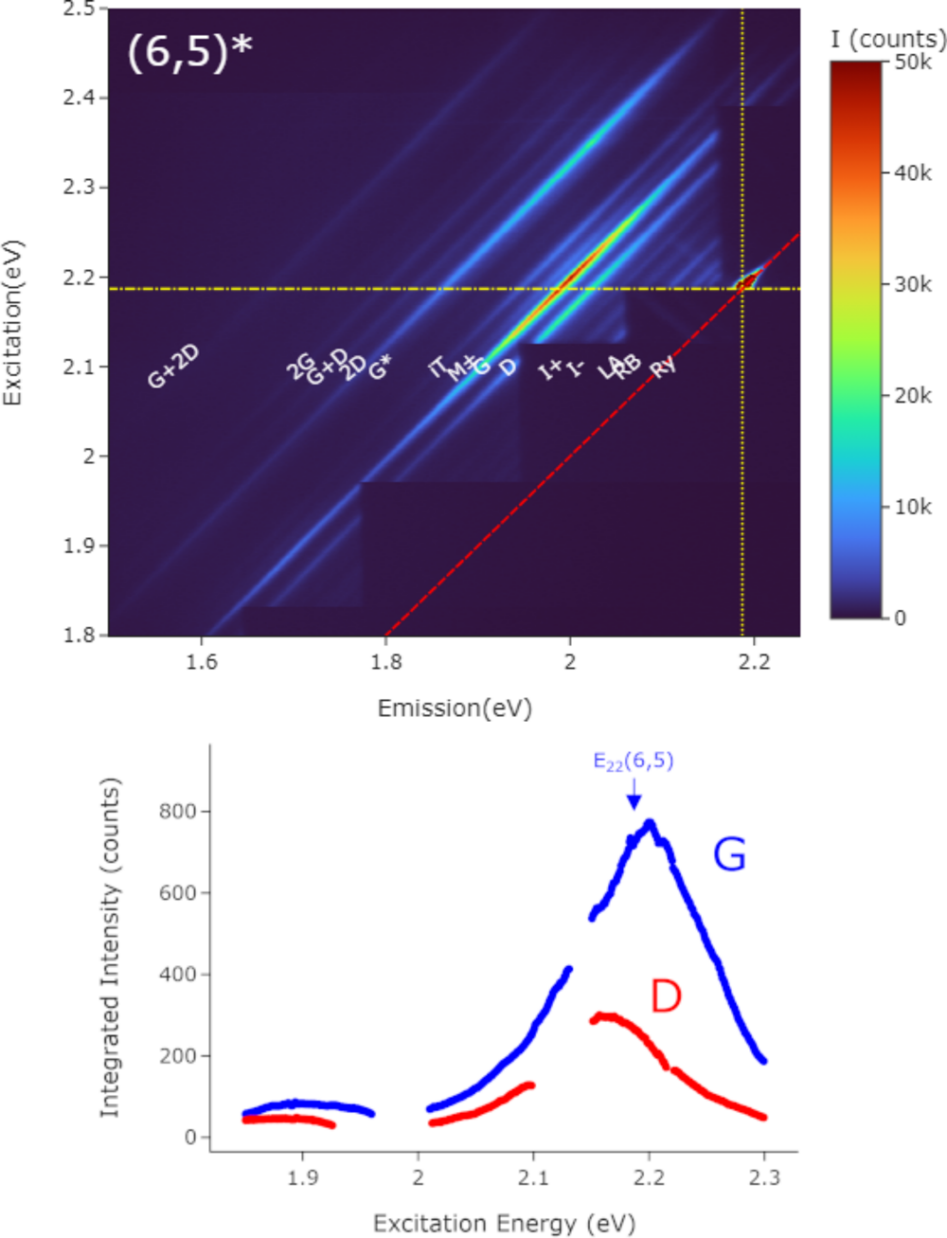
Raman excitation map for defective nanotubes. (top) Full spectrum Raman excitation map of a (6,5) nanotube with low crystallinity, and so a high D band. (The asterisk here just indicates that it is a low-crystallinity sample.) The lines and labels are as in Figure 5. (bottom) G and D band REPs extracted from the map. The breaks in the lines are where the fit is poor or impossible due to the filter edges. The expected position of the *E*_22_ resonance is labeled (see text).

Another commonly evaluated band is the D band, which is used to assess “defectiveness” in SWCNT samples. The samples presented above exhibited low D band intensities which complicated the experimental evaluation of resonance effects. Thus, to increase the intensity of this band, we prepared the same materials with additional tip sonication to introduce more defects into the crystalline lattice. Data from this type of sample are shown in Figure 6. The imaging conditions were exactly the same, as is the absolute intensity scale in the figure. The additional defect-induced SWNCTs show a reduction in G band intensity of almost 2×. In fixed-wavelength RS it is difficult to tell if intensity changes relate to shifts in the resonance energies or a change in overall scattering intensities. From an REM it is less ambiguous and we conclude that the change is not due to a shift but a reduction in intensity.

Focusing on the sample with increased defects in Figure 6, overall the D and G bands track each other, but the extracted D band REP maximum is noticeably lower than the G band in energy (∼50 meV). The G band mode originates from a simple single phonon scattering event, while the D band is commonly understood as a double resonance activated by disorder;^10,32^ therefore, a somewhat different perturbation term should be responsible for it. Recently there has been some controversy suggesting that the models used to explain graphitic carbon RS had diverged unnecessarily from common models used to explain RS in more general situations and questioning the sequence of scattering to and from real states for various RS bands in graphene and related materials.^33^ It is difficult to draw any conclusion from what is shown here alone, but the broadly similar appearance of the REPs could be consistent with models where the features have a common origin. That said, differences in the REP structures should help clarify the differences in their physical origins. Regardless, experimental REMs like these have the potential to shed light on this question and perhaps resolve it by including more of the physics than fixed-wavelength RS spectra, or of REPs of any one band on its own.

From a metrology viewpoint the difference in REP behavior has strong implications for quantitatively correct, intercomparable characterization of SWCNT samples. The D to G ratio, the integrated intensity of the D band to the G band, is frequently used as a metric for the crystalline defectiveness of SWCNT samples, as it is for all graphitic carbons. Clearly, however, due to resonance, this ratio is only meaningful up to some limit. The D and G bands are different *resonant* RS bands in SWCNTs and so *must* have different REPs. Thus, the D/G ratio intrinsically varies somewhat with resonant conditions. (Figure S11 in the Supporting Information). More widespread evaluations of REP behavior and intercomparisons are recommended to more fully account for this effect.

Finally, we show how the REM techniques in this work can address the fundamental question of the RS intensity for resonant materials like SWCNTs. For SWCNTs this is an important attribute that is challenging to constrain, with problems in quantification arising from its sensitive dependence on wavelength (and environment). Having the REM helps because it is clear where on the resonance enhancement curve the data come from. To illustrate the usefulness of REM, we evaluate the peak resonant RS scattering efficiency of the G band for the (6,5) on resonance, using a region of interest (ROI) analysis of the RS scattering to compare to the signal measured in straight reflectance. The results are outlined below, with additional details being given in the Supporting Information.

On the REM for the (6,5) we define a region of interest (ROI) 20 pixels high in excitation (corresponding to 18 meV width at 2.241 eV excitation energy, or 4.5 nm at 553 nm). For the G band we recorded 3.4 × 10^4^ counts/(s meV) as an integrated intensity—meV here refers to the excitation energy range. We also measured the elastically scattered light (“Rayleigh”) by reducing the integration time and using a neutral density filter of optical density 2 (10^–2^ transmission). At the same excitation energy, corrected for the filter, the elastic scattering of the (6,5) nanotubes was 5.8 × 10^8^ counts/(s meV). This means that the RS of the G band for (6,5) on resonance is 17000× weaker than the elastically scattered reflectance (“Rayleigh”). It is also interesting to determine how reflective the SWCNTs are at this wavelength or indeed as a function of wavelength (see the Supporting Information). For this, we used PTFE as a point of comparison. PTFE has a high (near-unity) and flat reflectance for visible wavelengths,^34^ such that measuring the reflectance from PTFE gives an indication of how much light is scattered (versus absorbed) by the SWCNT film. We recorded scattering of 1.5 × 10^11^ counts/(s meV), indicating that the (6,5) film (“Rayleigh”) scatters 1 out of 260 incident photons on resonance.

The ratio of 1 RS photon for 17000 Rayleigh scattered photons (∼6 × 10^–5^ efficiency), or in other terms a ratio of 1 RS photon for 4.4 × 10^6^ incident photons (∼2 × 10^–7^ efficiency), represents strong RS. By comparison, with a 514 nm laser, a monolayer of benzene will only scatter 1 RS photon in 6 × 10^14^ incident photons (∼2 × 10^–15^ efficiency) into a steradian (nearly the same collection angle as the objective here), and even a full volume of liquid benzene will only scatter 1 in 5 × 10^7^ incident photons (∼2 × 10^–8^ efficiency).^35^ There is little meaning to give an uncertainty for these values, as there is uncertainty in the exact transmission of the filter, reflectance of the PTFE, and response of the instrument at different wavelengths. The uncertainly in counts is very small, but a chain of calibrations is required to make measurements photometric. However, it does point a way toward photometric quality measurements of absolute resonant RS cross sections. Moreover, it shows in principle how an REM can eliminate some of the uncertainty that arises in fixed-wavelength RS experiments. We expect more rigorous and systematic absolute resonance RS intensity evaluations in the future.

Comparison to other materials can also provide insights about RS scattering intensities (Supporting Information). The G band of the SWCNTs here is ∼4000–10000× higher than the G band of graphite (HOPG). For the (8,3) nanotube, the highly resonant M band at its peak is ∼4000× greater than the graphite G band. Again, these multipliers should not be viewed as definitive because there are many sources of error (tube density, alignment/polarization, the high background relative to HOPG, spectral characteristics of neutral density filters, etc.). The important point is that this kind of data analysis is a pathway to being more quantitative about RS intensities.

Raman excitation mapping of the RBM with a tunable laser is an established method to determine nanotube (*n*,*m*) because of the strong diameter dependence of that mode and the narrow resonant line width.^2,3,5,7^ It may be interesting to ask whether (*n*,*m*) can be determined from larger Raman shift modes like the ones we track here. The answer is definitely “yes”, at least in principle. Here, the energy of the *E*_22_ optical resonance can be determined from higher Raman shift modes. Modes other than the RBM also have Raman shifts which change with diameter, for example the G^–^ band,^13^ which changes, though less prominently than the RBM mode. For REM to be most effective as an assignment tool, the resolution should be high and the Raman shift calibration should be precise. There is a great deal of information in the REM “QR-code” that we cannot yet read in detail. Every vibrational mode will have its own species-dependent intensity which has its own REP, though it depends at least to some small extent on extrinsic factors like the environment. Some of this REP intensity variation is already clearly visible in the map we have shown here, but untangling the extent to which the full REM can be used for characterization will need more experimental and theoretical work in the future.

Another exciting aspect for the future is to investigate the differences in REPs. For the same (*n*,*m*), the G band REPs extracted here are broadly similar to prior work, but there are clear systematic differences, particularly in the broadening term (Γ). This is an interesting and likely informative dimension to explore even with the more common tunable Raman spectroscopy systems. A particularly interesting aspect is the quantum interference effect that shapes the REPs in the maps. Because FS-REM is relatively fast and fairly simple, it could make such data more accessible to the community.

## Conclusion

To summarize, FS-REM was used to map out the variations in many Raman bands for three chirally pure SWCNTs, the (6,5), (7,5), and (8,3) species, over broad ranges of excitation and emission energies corresponding to excitation wavelengths from the blue (∼480 nm) to the NIR (∼740 nm) and emission wavelengths from the green (∼540 nm) to the NIR (∼950 nm). This captured whole series of RS bands and their REPs, including D (strong for low crystallinity only), G, and 2D bands and also less studied bands including LA, IFM, M, iTOLA, G*, G+D, 2G, and G+2D. Both RS and PLE were captured on the same detector for the (8,3) species. The structure of the REMs can be interpreted in terms of an elementary picture including ingoing and outgoing resonances; however, the real structure is complicated and each band has its own profile. Lower crystallinity samples with additional induced defects showed a reduced overall scattering intensity. In the case of low-crystallinity (6,5) SWCNTs the D band profile tracks the G band to some extent but the profiles are shifted. Future observations of this kind may directly address the interesting controversy in the theory behind the origins of such bands. By measuring RS—and reflectance (“Rayleigh”)—and comparing to a standard like PTFE or HOPG, it is possible to determine RS scattering intensities without the complication that resonant RS is wavelength dependent.

## Methods

Certain commercial equipment, instruments, or materials are identified in this paper in order to adequately specify experimental details. Such identification does not imply recommendation or endorsement by the National Institute of Standards and Technology (NIST) or by the National Research Council Canada (NRC), nor does it imply that the materials or equipment are necessarily the best available for the purpose.

The optical setup is illustrated schematically in Figure 1. The supercontinuum light source (SC) was an NKT Photonics SuperK Extreme Super Continuum White Light Laser (EXR-15). This emits wavelengths from ∼480 to ∼2 μm. A cleanup filter (CF) stage blocks unwanted infrared light. Six sets of filter pairs were used (four sets only for Figure 2), each pair comprising an excitation filter (ExF) and emission filter (EmF). The excitation filters were short wave pass filters, and the emission filters were long wave pass filters. Each pair had a different cutoff (nominally 532, 564, 600, 633, 700, 750 nm). For Figure 2 only four of these were used. A 300 lines/mm (lpmm) transmission excitation grating (ExG) was used to provide a large bandwidth (i.e., a wide range of wavelengths). A nominally 50:50 plate beamsplitter was used. A 20× infinity-corrected long working distance objective was used to focus the illumination into a rainbow line on the sample (S) and collect the scatter. Beam dumps (BD, BD1, BD2) collected unwanted scatter. The emission grating (Gr) was a 300 lpmm transmission grating. A 100 mm tube lens (TL) was used to focus the light on a cooled sCMOS camera (Andor Neo). The wavelengths of emission and excitation were calibrated with the use of band-pass filters. For Figure 2. 30 s integrations were used, with a sum of three 10 s integrations. For the other REMs shown in the paper, 12 s integrations were used, by summing 6 integrations of 2 s each. A data set was taken for each of the filter pairs and combined to make a single map.

To produce the (6,5) and (7,5) chirality-pure SWCNTs, the source material was a cobalt–molybdenum catalyst (CoMoCat) SG65i purchased from Sigma-Aldrich (Cat. #773735). For the (6,5) nanotubes the wrapping polymer was poly[(9,9-dioctylfluorenyl-2,7-diyl)-*alt*-co-(6,6′-{2,2′-bipyridine})] (PFO-BPy6,6′) purchased from American Dye Source Inc. (*M*_w_ 34 kDa, polydispersity 4.3). For the (7,5) nanotubes the wrapping polymer was poly(9,9-di-*n*-octylfluorenyl-2,7-diyl) (PFO), synthesized in NRC laboratories (*M*_w_ 54 kDa, polydispersity 2.4). For both (6,5) and (7,5) pure materials, 15.6 mg of the SWCNT source was mixed with a suitable wrapping polymer (15.6 mg) in 25 mL of toluene.^36^ The mixture was tip-sonicated (Branson Sonifier 250) with a minitip of ∼5 mm (3/16 in.) at an output of 30% and a duty cycle of 60% for 30 min, followed by centrifugation at 1310 rad/s (12500 rpm) for 60 min (SS-34 rotor, a relative centrifugal force of 18700*g*, *g* ≡ 9.81 m/s^2^). The enrichment was repeated for multiple cycles to maximize the mass yield.^18^ The enriched (6,5) and (7,5) were previously found to have an increasing number of defects as the total sonication time was increased.

To produce the (8,3) chirality-pure SWCNTs a previously reported ATPE method^19,20,37^ was used. The CoMoCat process synthesized SWCNTs (Chasm Nanotechnologies, SG65i grade, lot 64) were used as source materials for a dispersion. Briefly, rate-zonal centrifugation purified SWCNTs,^2^ initially in a 10 g/L sodium deoxycholate (DOC) solution, were mixed into a spontaneously separating mixture of two water-soluble polymers, polyethylene glycol (PEG, 6 kDa, Alfa Aesar) and dextran (Dextran 70, TCI America). By adjusting and controlling the concentrations of DOC, sodium dodecyl sulfate (SDS), sodium cholate (SC), and sodium hypochlorite across multiple ATPE extractions,^20,37^ an aliquot greatly enriched in the (8,3) species was obtained. Repeated concentration and dilution in a stirred ultrafiltration cell (Millipore) was used to remove the polymers used in the ATPE to concentrations <0.1 μg/mL and to set the DOC concentration to 10 g/L in H_2_O.

The enriched (6,5) and (7,5) dispersions were filtered through PTFE membranes (pore size 0.2 μm) by gravity (without vacuum), and the films were rinsed with plenty of toluene to remove excess polymers. The films on the membranes were mounted on glass slides and used for Raman measurement. For the (8,3) nanotubes, an optically thick spot was prepared by repeatedly drop-casting on a CaF_2_ glass substrate (Raman grade, Crystran Ltd.) The aqueous dispersion was hand-sampled by a micropipet. Several μL of liquid was deposited and allowed to dry, repeatedly, in the same place, until an opaque spot a few mm in diameter formed at the center. This gave excellent signal strength and proved to be adequately uniform. However, moving near the edges of the spot caused distortions in the shape of the line illumination, so maps were taken only from the center of the spot.

## Supporting Information Available

Illumination profile, photoluminescence excitation maps, and region of interest (ROI) analysis of scattering intensities, response calibration with respect to graphite, response calibrated G and D bands, D/G ratios, narrow M and iTOLA band profiles (PDF)

## Abbreviations

SWCNT: single-walled carbon nanotube
RBM: radial breathing mode
RS: Raman scattering
REP: Raman excitation profile
REM: Raman excitation map
FS-REM: full spectrum Raman excitation map
PL: photoluminescence
PLE: photoluminescence excitation
ROI: region of interest
Ry: Rayleigh
ATPE: aqueous two-phase extraction
