# Supplementary material for: Broadband Full-Spectrum Raman Excitation Mapping Reveals Intricate Optoelectronic–Vibrational Resonance Structure of Chirality-Pure Single-Walled Carbon Nanotubes
Source: ACS Nano. 2023 Apr 3;17(8):7285–95. doi: 10.1021/acsnano.2c10524 (PMC10134487; doi:10.1021/acsnano.2c10524)
Supplement: Supplementary file 1 — nn2c10524_si_001.pdf [file nn2c10524_si_001.pdf]

**Supporting Information for**

# Broadband full-spectrum Raman excitation mapping reveals intricate optoelectronic-vibrational resonance structure of chirality pure single walled carbon nanotubes

*Paul Finnie[†], Jianying Ouyang[†], Jeffrey A. Fagan[§]*

[†]National Research Council Canada, 1200 Montreal Road, Ottawa, Ontario, K1A 0R6, Canada
[§]Materials Science and Engineering Division, National Institute of Standards and Technology (NIST), Gaithersburg, MD 20899, USA.

*Certain commercial equipment, instruments or materials are identified in this paper in order to adequately specify experimental details. Such identification does not imply recommendation or endorsement by National Institute of Standards and Technology (NIST) or by the National Research Council Canada (NRC), nor does it imply that the materials or equipment are necessarily the best available for the purpose.*

**Illumination Profile**

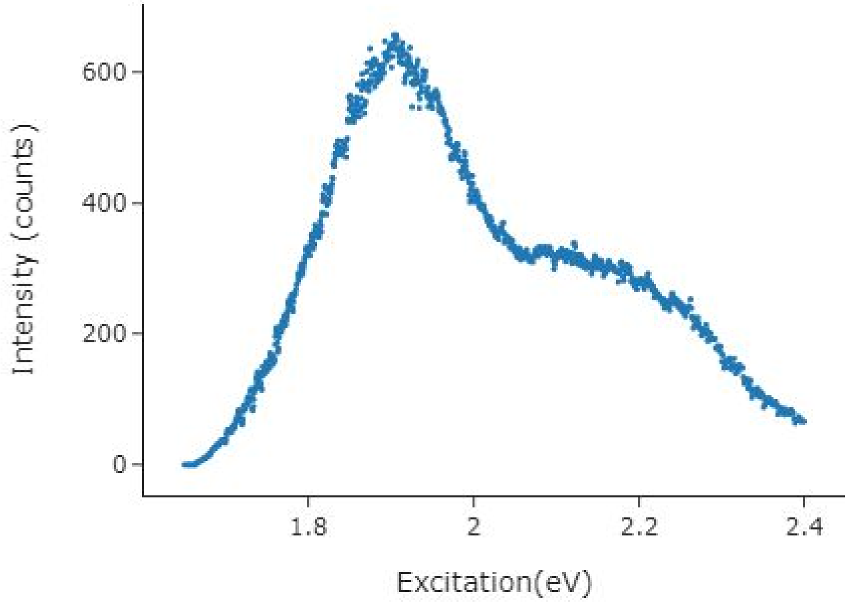


**Figure S1**. Illumination Intensity Profile

The spectrum of supercontinuum light dispersed chromatically into the "rainbow line" and reflected off a flat polytetrafluoroethylene (PTFE) surface using the same full spectrum Raman excitation mapping (REM) setup as above but with the excitation and emission filters removed and a neutral density filter only (ND3.0, 0.001× power) to reduce the excitation power. The illumination line was fit by a single Lorentzian and its amplitude extracted to give integrated intensity. The integration time was 500 μsec.

**Photoluminescence Excitation Maps**

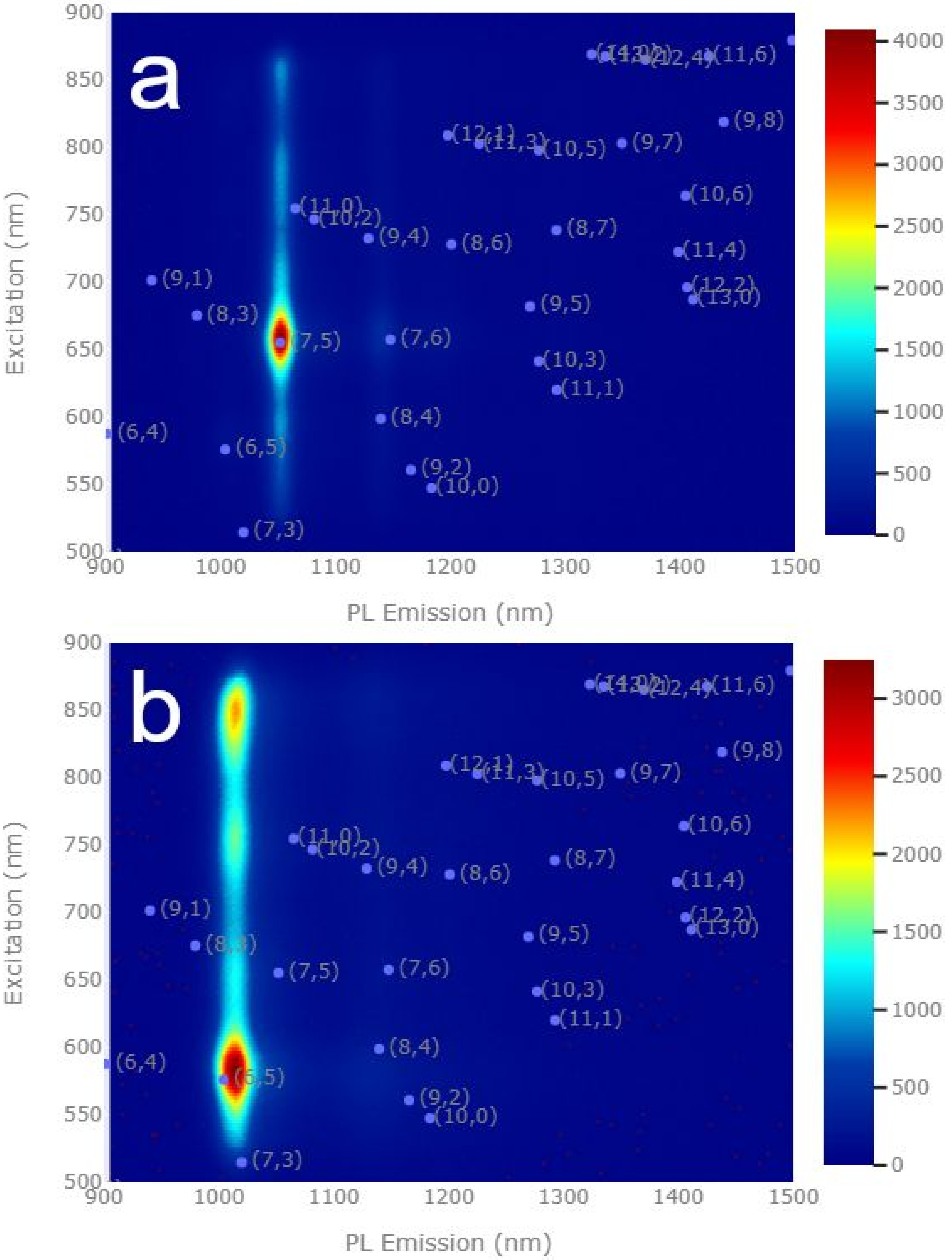


**Figure S2.** Photoluminescence Excitation Maps of Polymer Wrapped (a) (7,5) Nanotubes and (b) (6,5) Nanotubes

Examples of PLE maps of sorted polymer wrapped SWCNTs of the types used to make the films in the main text, but in toluene suspensions. Labels of (n,m) indices are from ref [S1]. A home built real-time PLE system similar to Ref. [S2] was used to obtain these maps, with a setup as described in Ref. [S3]. Solutions were measured in quartz capillaries. The total acquisition time for the map was 1.0 msec for (a) and 1.2 msec in (b). The solid films of these two emitted photoluminescence (not shown) but did not show well-structured PLE features.

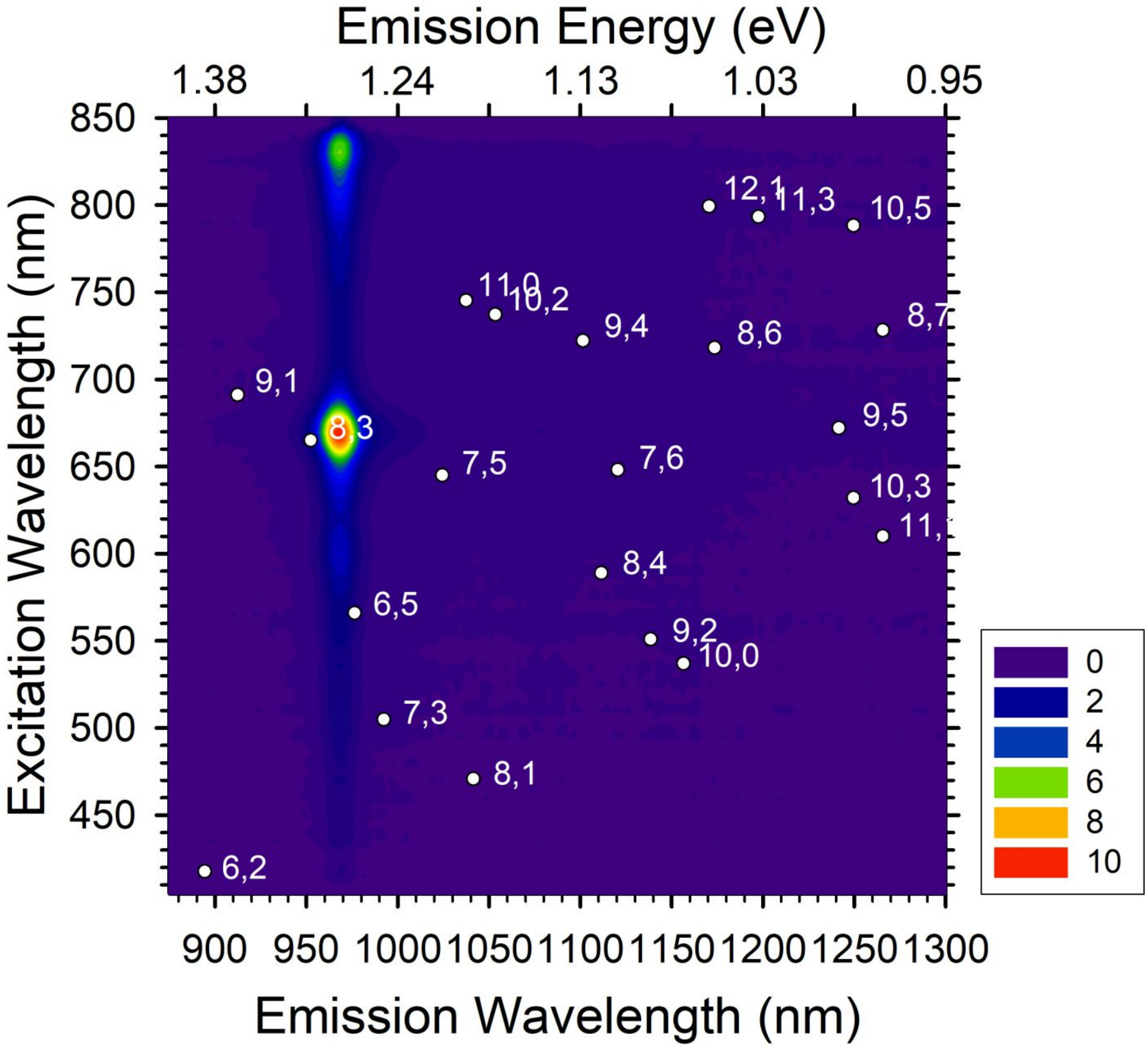


**Figure S3**. Photoluminescence Excitation Map of (8,3) Nanotubes

Fluorescence of the ATPE sorting-based surfactant wrapped (8,3) SWCNT sample [S4,S5,S6] was measured in a heavily diluted aliquot for minimal inner-filter effects in 10 g/L DOC in $H_2O$ at room temperature. Two-dimensional excitation *versus* emission NIR fluorescence was collected in a NS-SuperChirooptical instrument (Applied NanoFluorescence) from 405 nm to 850 nm in excitation with a 5 nm bandpass and 5 nm step in a back collection geometry with acquisition settings of 3 x 800 ms collection at each step. Tabulated data [S1] is used to produce the species labels shown on the plot. The structured (8,3) nanotube PLE was also clearly visible for the solid films (see main text).

## Comparison of Raman Scattering Intensity to “Rayleigh” Scattering Intensity

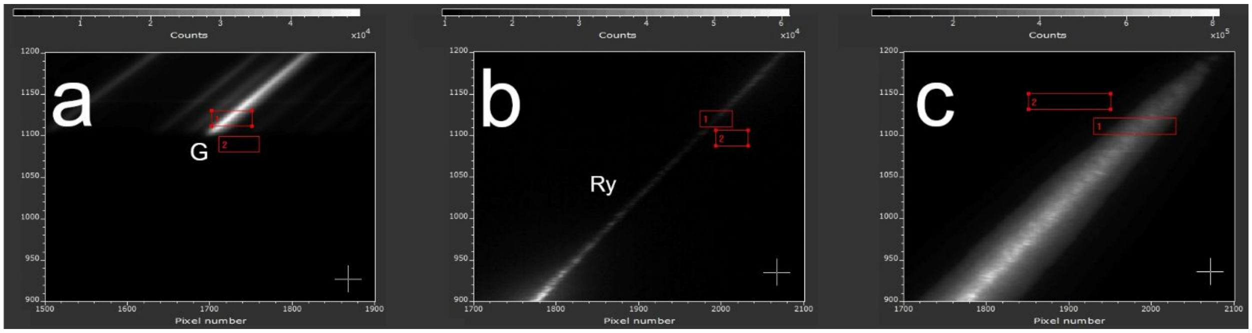


**Figure S4.** Scattering Intensity Region of Interest (ROI) Analysis: (a) Raman scattering of (6,5) nanotubes with filters. (b) Reflectance (“Rayleigh scattering”) of (6,5) nanotubes. (c) Reflectance (“Rayleigh scattering”) of PTFE.

The vertical axis is the supercontinuum laser energy axis in all three panels (in pixels). The horizontal axis is the scattered light energy axis in (in pixels). One region of interest (ROI) box is used to sum the scattered light signal. This box was chosen to be centred at 2.24 eV with a height equivalent to 18 meV (corresponding to 556 nm centre, height of 4.5 nm). A second box of the same size nearby is used as the background. Frame (a) shows the raw signal on the camera for (6,5) nanotubes with excitation and emission filters in place. The G band is labelled and is on or near to resonance. Three 10s integrations were summed for a 30s total integration time. Frame (b) shows the same (6,5) SWCNTs in the same area with the edge and emission filters removed and a neutral density filter of optical density (OD) 2.0 on the excitation side. 100 integrations of 102 µsec were added. Frame (c) shows the same illumination profile, but on a PTFE surface. PTFE has a flat reflectivity profile versus energy (or equivalently wavelength), with a reflectance near unity. (The position of the illumination line on the sample and on the camera was different here than for the other data shown in the paper.)

| Material | Setup | Filters | Signal (count) | Bkgd. (count) | Net (count) | Time (s) | Filter factor | Net (counts/sec) |
|---|---|---|---|---|---|---|---|---|
| (a) (6,5) | Raman (G band) | Edge filters | $2.0\times10^{7}$ | $1.3\times10^{6}$ | $1.8\times10^{7}$ | 30 | 1× | $6.2\times10^{5}$ |
| (b) (6,5) | Reflectance ("Rayleigh") | Neutral Density (OD2.0) | $9.7\times10^{6}$ | $8.6\times10^{6}$ | $1.1\times10^{6}$ | 0.0102 | 100× | $1.1\times10^{10}$ |
| (c) PTFE | Reflectance ("Rayleigh") | Neutral Density (OD2.0) | $3.2\times10^{8}$ | $4.2\times10^{7}$ | $2.8\times10^{8}$ | 0.0102 | 100× | $2.8\times10^{12}$ |

**Table S1**. Scattering efficiency estimation.

The scattering intensity is quantified for each of the three panels. Row (a) is (6,5) SWCNTs with edge filters in place for Raman excitation mapping. The integrated G band intensity in counts and background is given for the box in Figure S3.  Row (b) shows the (6,5) signal from the same area with no filters other than a neutral density (OD2.0) filter on the excitation side. It therefore is the reflectance, or loosely speaking, "Rayleigh scattering" of the SWCNTs. (The term Rayleigh scattering is not strictly correct for a dense film such as this.) Row (c) shows the signal for the same illumination profile but on bare PTFE. The signal is the integrated intensity in counts for the boxes with strong signal in Figure S3. The background is the integrated intensity in counts for the no-signal areas of the same panels. Net is the signal less the background in counts. The time is the total integration time. The filter factor is the correction factor to take into account the filter transmission, a factor of 100 for the OD2.0 filter. The corrected net signal in counts per second. The (6,5) reflectance signal is ~260× lower than the PTFE. The (6,5) G band Raman scattering signal is ≈$1.7\times10^{4}$× less than its reflectance at this resonance.

**Calibration of Raman Scattering Intensities**

The scattering intensity is proportional to the incident power and the response of the system. One common and relatively simple way to deal with that is to use a known sample and determine correction factors based on that sample. Here, highly oriented pyrolytic graphite (HOPG) (SPI Supplies, Ltd.) was used as the comparison sample. The G and 2D (commonly called G') bands can be seen, over the background. An unprocessed HOPG REM with one filter set with a 633 nm edge is shown below. (Figure S5)

The HOPG signal is much weaker than that of the SWCNTs, and the G and 2D of graphite were difficult to see against the background. To make the Raman bands more visible, the REM was coarsened by averaging over windows of 8 pixels square and subtracted from a rigidly 12 pixel shifted "rolled" copy. (Figure S6)

The 2D band was fit with a Lorentzian function over the entire excitation range and a polynomial was fit to the extracted intensity. (Figure S7) The G band was fit over a limited range. The ratio of G to 2D band intensities was found to be fairly close to unity. (Figure S8). So it is reasonable to use the same correction factor for the G band as well.

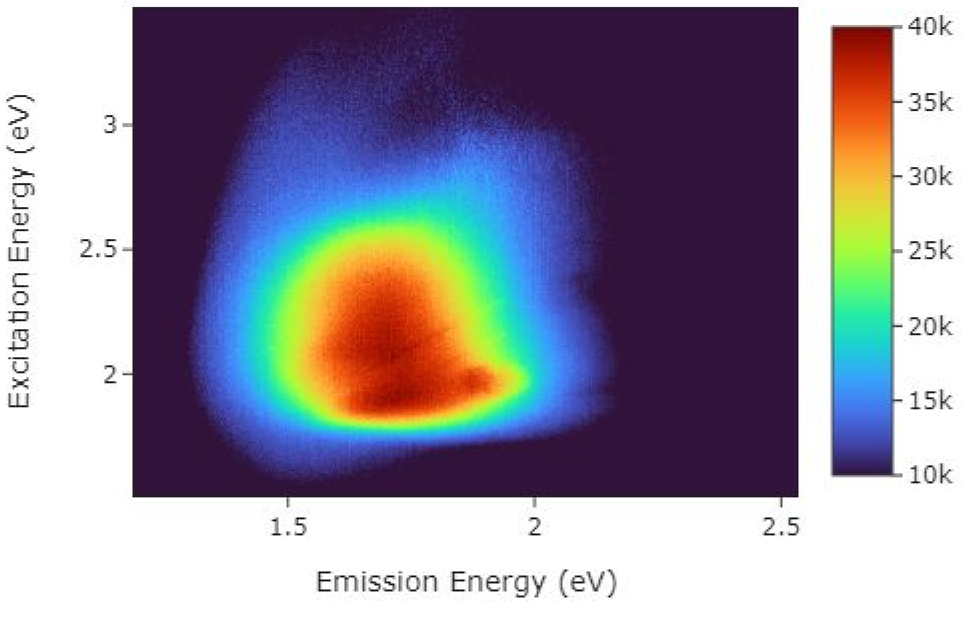


**Figure S5**. Raw Raman Excitation Map of HOPG

The color scale of the intensity is in instrument counts. The integration time was 60 seconds. A neutral density filter (ND10, 0.1× power) used for the SWCNT samples was removed to increase the incident power and thus the scattered light intensity. This map was taken with filter sets for 633 nm edges.

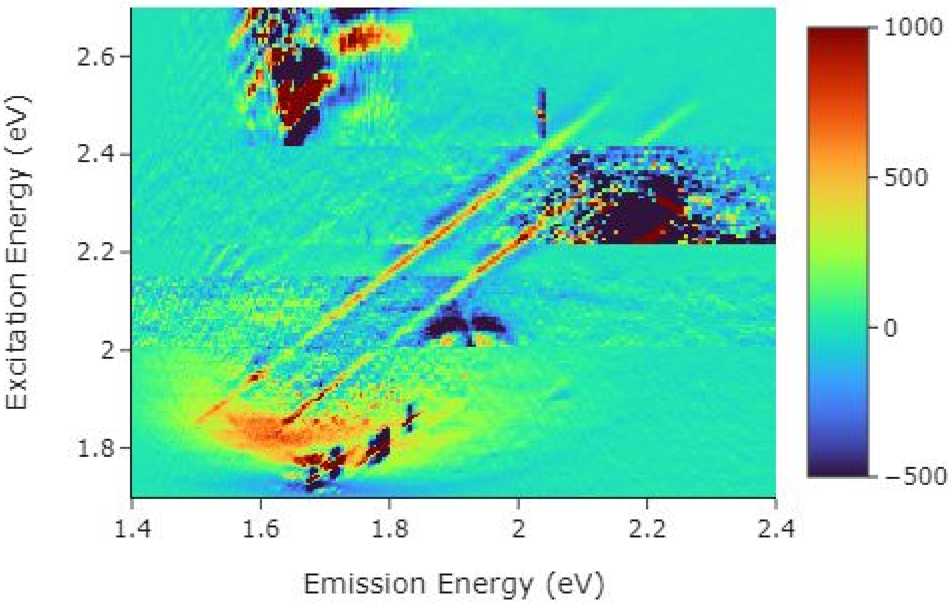


**Figure S6**. Background Corrected REM of HOPG

The HOPG intensity for all filter sets after compensating for the weak signal and strong background by coarsening the two-dimensional data and subtracting a shifted “background” region from the same map. The red diagonal line above the centre is the 2D band, while the lower one is the G band. Ripples and other features are artifacts of the background and the subtraction process.

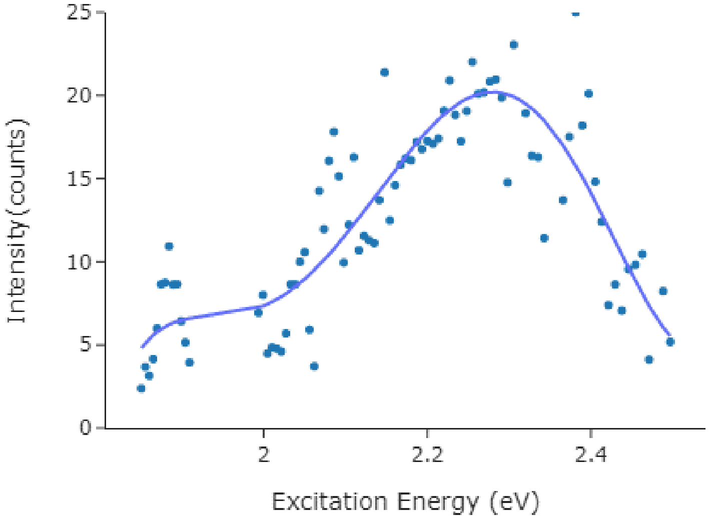


**Figure S7**. HOPG 2D Band Intensity

The intensity of the 2D Raman band determined by the amplitude of a Lorentzian fit across the probed excitation energy range. The line is a polynomial fit that reasonably approximates the

combination of illumination and detector response. Data between 1.9 and 2.0 eV was discarded for the fit due to the large noise fluctuations. The intensity in SWCNT sample measurements can be divided by this polynomial to take into account the variation in illumination intensity and detector response.

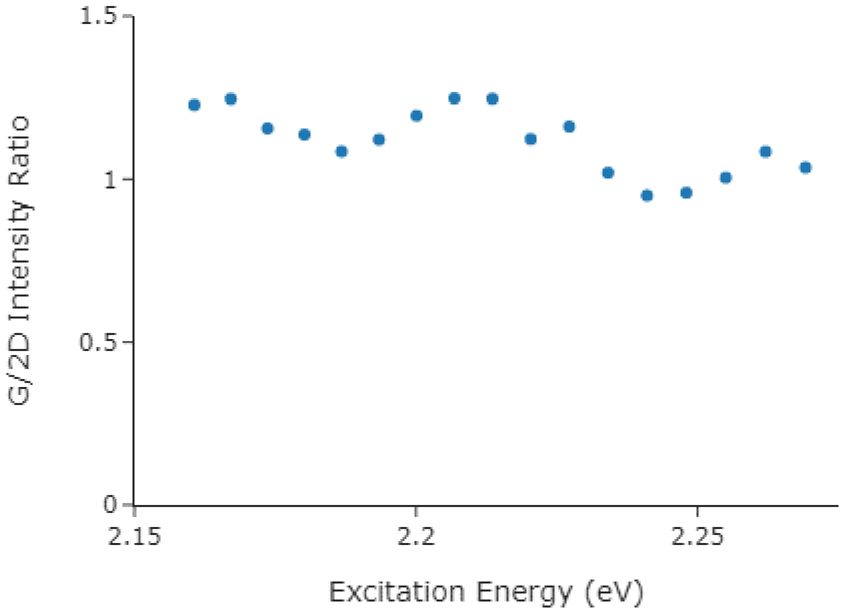


**Figure S8**. Ratio of Intensities of the G Band and 2D Bands for the HOPG Sample

A similar data set to Figure S7 can be calculated for the G Band signal from the HOPG. Greater fluctuations in the background for the G band cause this signal to be noisier than the 2D data. However, the G and 2D (also called G' for graphite) could both be extracted over a limited range of energies. The ratio of the two intensities over this excitation range is plotted here. The ratio is close to unity.

## Corrected Band Intensities

With the simple polynomial in excitation modeling the effect of illumination intensity and detector response, corrected band intensities can be calculated and are plotted in Figure S9. The corrected G band intensities for the three SWCNT species further include a correction factor of 10×, coming from the ND10 excitation filter that was used for the SWCNTs, and a correction factor corresponding to the shorter integration time (For HOPG 60 seconds integration was used, while SWCNTs the integration was only 12 seconds).

The SWCNT signal is ≈5000x more intense than the signal from HOPG. For these samples we observe that the $E_{22}$ + G band resonance is weaker than the $E_{22}$ resonance, and that they overlap significantly. The ≈2× difference in intensity of the ingoing and outgoing energies is similar to those of G band resonant excitation profiles for $E_{22}$ previously reported. [S7]

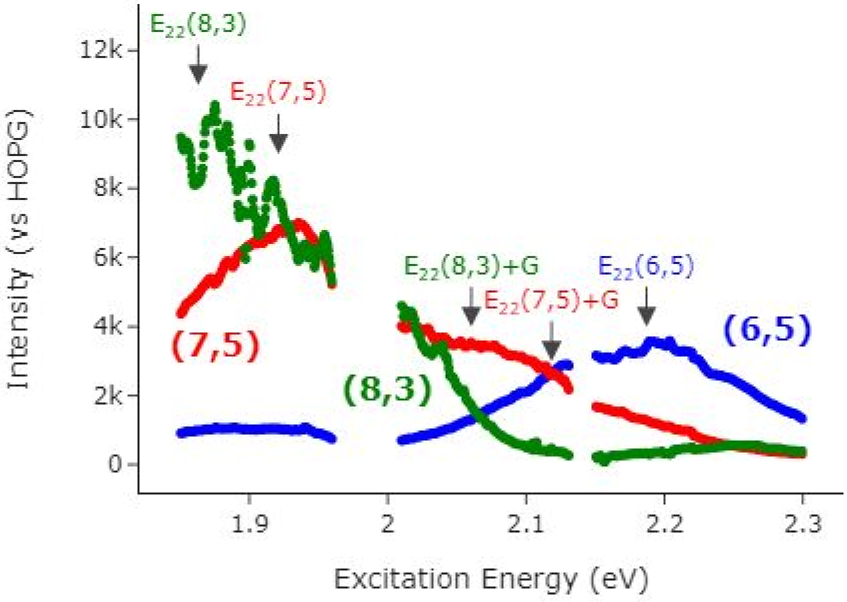


**Figure S9**. Intensity Corrected Raman Excitation Profiles

The G band intensities of Figure 5 in the main text are replotted by correcting them with polynomial fit to the HOPG response, and correcting for the higher power used for HOPG, and the longer integration time. Non-resonant Raman scattering would be expected to yield a flat, horizontal line.

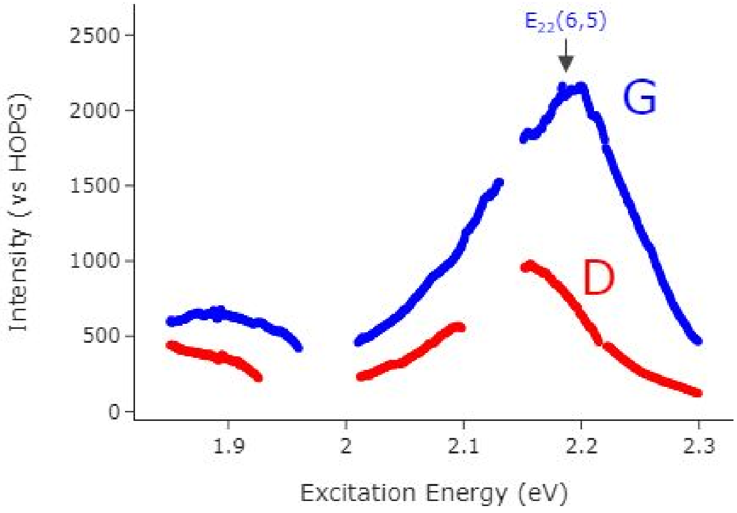


**Figure S10.** Corrected G and D bands

The D and G band intensities for the low crystallinity (6,5) nanotube of Figure 6 in the main text are replotted with correction as in Figure S9.

**D/G Ratio Variation**

A consequence of the D and G bands having slightly different peaks is that the D to G ratio varies with excitation energy. This is shown in Figure S11 for the low crystallinity (6,5) nanotube sample.

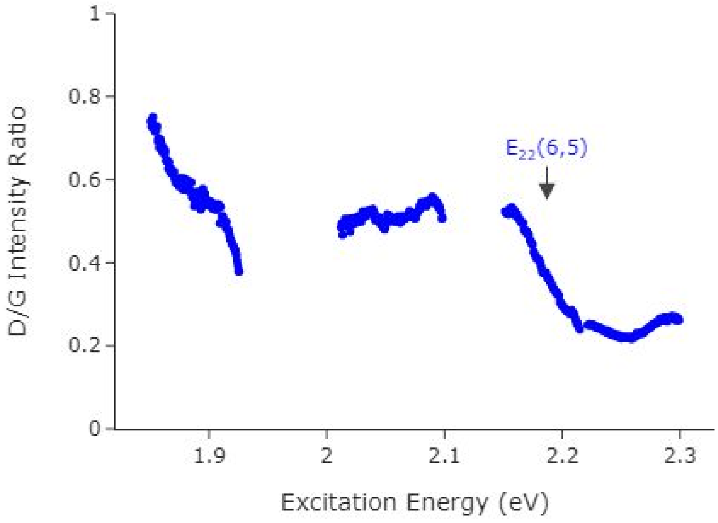


**Figure S11.** D/G Ratio

The ratio of integrated intensities of the D and G bands for the low crystallinity (6,5) nanotube sample used in Figure 6 in the main text.

## Narrow and Strong Resonant Excitation Profiles for Some Bands

The Resonant Excitation Profile (REP) of the M band of the (8,3) sample was narrow compared to the G band REP (M+ and M- are not separately resolved). The iTOLA band (abbreviated iT in the plot) band was similarly narrow. For the (6,5) SWCNT the iT band was likewise narrower than the G.

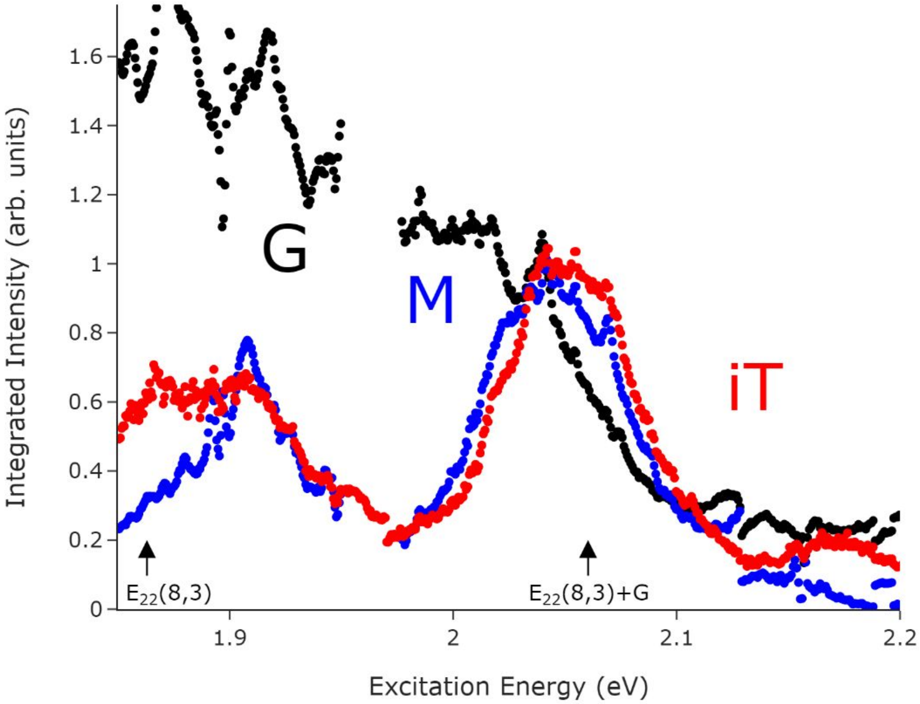


**Figure S12.** Normalized Resonant Excitation Profiles of the G, M and iTOLA bands for the (8,3) nanotube.

The uncorrected Resonance excitation profiles (REPs) of the G, M and iTOLA bands (iTOLA is labelled iT in the graphic) are shown for the (8,3) sample. They have been scaled arbitrarily so that they have similar values near the peaks of the M and iT bands. This normalization makes it easy to compare their widths. The G and M band intensities were obtained by fitting the two bands simultaneously with two Lorentzians. The iT band was fit separately as a single Gaussian peak. The sharp fluctuations in the low energy range (≈1.9 eV) are due to the photo-instability of this sample which was pronounced at these powers for excitation energies near the $E_{22}$ resonance. The REP peak near 2.04 eV for the M band and the peak near 2.05 eV iT band are both steady at this illumination intensity, however. As can be seen the M and iT bands are much sharper than the G band. As apparent in the map of Fig. 4 in the main text, before normalization, for the (8,3) SWCNT, there is a region where the M band is on resonance and its integrated intensity actually exceeds the G band intensity. This is unchanged by HOPG normalization.

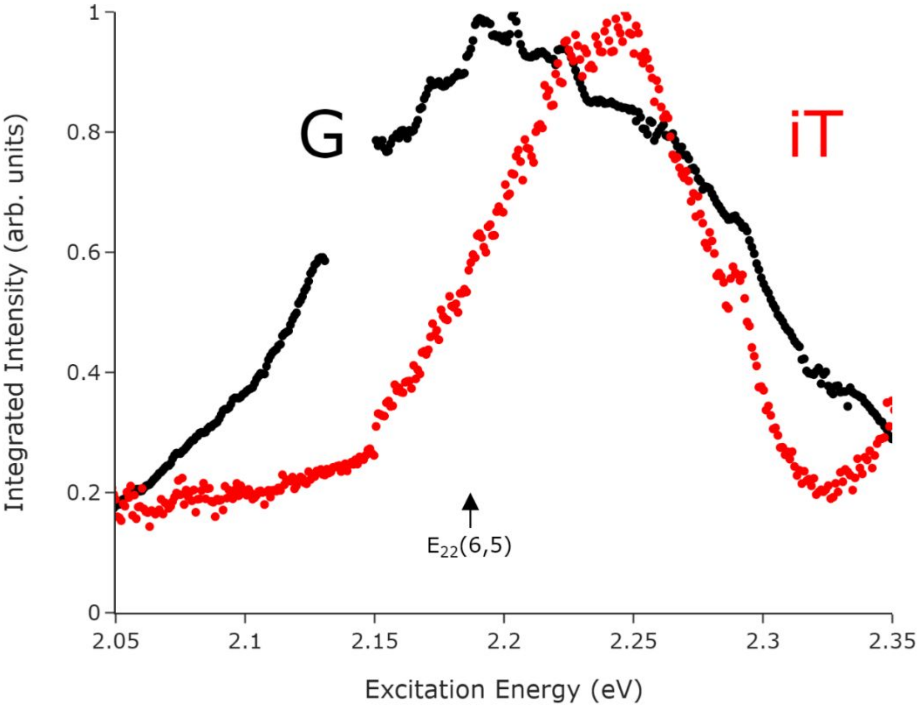


**Figure S12.** Normalized Resonant Excitation Profiles of the G and iTOLA bands for the (6.5) nanotube

The uncorrected Resonance excitation profiles (REPs) of the G and iTOLA bands (labelled iT in the graphic) for the (6,5) sample are shown. The G band was fit with a single Lorentzian and the iTOLA band was fit with a single Gaussian. To better compare their widths the resonant excitation profile peaks are scaled so that for both the maximum is equal to one. The iTOLA band is narrower than the G band for the (6,5) SWCNT.

**References**


[S1] R.B. Weisman, S.M. Bachilo, Dependence of optical transition energies on structure for single-walled carbon nanotubes in aqueous suspension: An empirical Kataura plot, Nano Lett. 2003, 3, 1235-1238.

[S2] Lefebvre, J. ACS Nano, Real time hyperspectroscopy for dynamical study of carbon nanotubes. *ACS Nano* 2016, 10, 9602–9607.

[S3] Ouyang, J.; Shin, H.; Finnie, P.; Ding, J.; et al. Impact of Conjugated Polymer Characteristics on the Enrichment of Single-Chirality Single-Walled Carbon Nanotubes. ACS Applied Polymer Materials 2022, 4, 6239 – 6254.

[S4] J.A. Fagan, C.Y. Khripin, C.A. Silvera Batista, J.R. Simpson, E.H. Haroz, A.R. Hight Walker, M. Zheng, Isolation of specific small-diameter single-wall carbon nanotube species via aqueous two-phase extraction, Adv. Mater., 2014, 26, 2800-4.

[S5] J.A. Fagan, Aqueous two-polymer phase extraction of single-wall carbon nanotubes using surfactants, Nanoscale Adv. 2019, 1, 3307-3324.

[S6] H. Gui, J.K. Streit, J.A. Fagan, A.R. Hight Walker, C. Zhou, M. Zheng, Redox sorting of carbon nanotubes, Nano Lett. 2015, 15, 1642-6.

[S7] G. Gordeev, B. Flavel, R. Krupke, P. Kusch, S. Reich, Asymmetry of resonance Raman profiles in semiconducting single-walled carbon nanotubes at the first excitonic transition, Phys. Rev. B 2019, 99 045404-1 -- 045404-6
